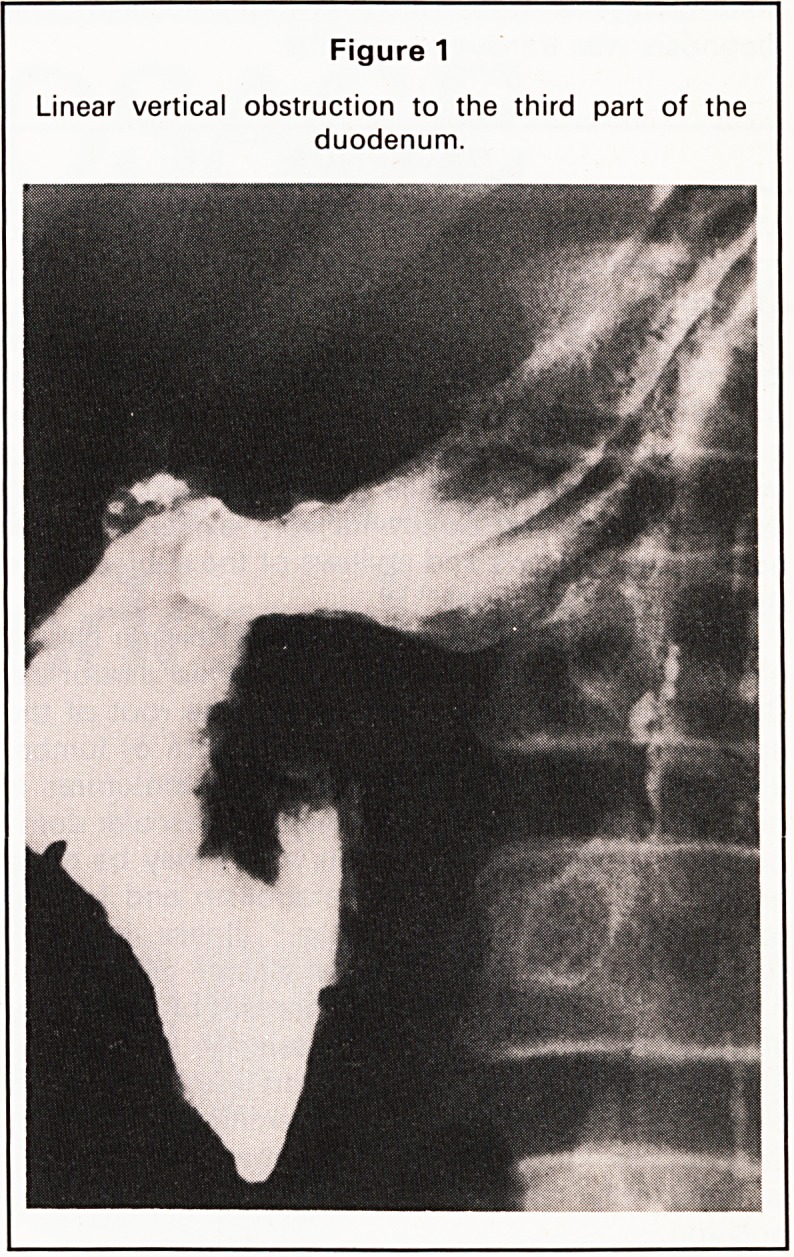# Vascular Compression of the Duodenum, Operative and Non-Operative Treatment

**Published:** 1984-10

**Authors:** A. P. Corfield, R. E. May

**Affiliations:** Department of Surgery, Frenchay Hospital, Bristol; Department of Surgery, Frenchay Hospital, Bristol

## Abstract

Two cases of acute vascular compression of the duodenum are described. Enteral feeding via a small bore nasojejunal tube is suggested as a safe and effective method of treatment in suitable cases.


### Vascular Compression of the Duodenum — Operative and Non-operative Treatment

**A. P. Corfield, F.R.C.S.**
Registrar
**R. E. May, M.S., F.R.C.S.**
Consultant
Department of Surgery, Frenchay Hospital, Bristol

**SUMMARY**

Two cases of acute vascular compression of the duodenum are described. Enteral feeding via a small bore nasojejunal tube is suggested as a safe and effective method of treatment in suitable cases.

**INTRODUCTION**

Vascular compression of the duodenum requiring medical intervention is an uncommon and controversial entity. Compression may be chronic with intermittent exacerbations or may present acutely following severe loss of weight. We report two acute cases and suggest an alternative line of conservative treatment.

*Case 1*
A 21-year-old female social worker was admitted with a 2-day history of severe abdominal colic and vomiting which had followed ingestion of a large meal. She had always been thin and had recently lost 10 kg weight, which she attributed to examination stress. On examination she was emaciated with a large tender tympanitic swelling which occupied the left side of the abdomen. Plain abdominal radiograms showed a dilated stomach and duodenum with the characteristic 'double bubble' of distal duodenal obstruction; nasogastric aspiration obtained 2600 ml of bile-stained fluid. A barium meal defined a linear vertical obstruction to the third part of the duodenum (Figure 1). At laparotomy the stomach and duodenum were found to be dilated proximal to the superior mesenteric artery, which compressed the underlying duodenum against the spine. Gastrojejunostomy was performed, and the patient made an uneventful postoperative recovery. She gained weight rapidly but has subsequently suffered intermittent gastritis related to bile reflux.

**Figure 1**

Linear vertical obstruction to the third part of the duodenum.

*Case 2*
A 13-year-old boy was admitted for neurosurgical assessment. Two weeks previously he had developed back pain, lower limb parasthesia and weakness. He was initially thought to have viral meningitis and was confined to bed. Vomiting began insidiously but progressed until he was unable to retain any food or drink. On examination he was very thin with a distended epigastrium and succussion splash. Plain abdominal X-rays showed a dilated stomach and duodenum. A barium meal revealed a vertical extrinsic obstruction to the third part of the duodenum; mesenteric vascular occlusion was diagnosed. A conservative management policy was adopted in view of his unexplained neurological signs and, as back pain precluded the prone position, a 110 cm transnasal enteral feeding tube (Viomedex) was passed. Under fluoroscopic control the tube was negotiated through the compressed duodenum until the tip was seen to be in the jejunum. High protein and calorie tube feeding (Isocal) was instituted. He stopped vomiting and 5 days later oral feeding was started. He progressed rapidly and has subsequently made an uneventful recovery. The final neurosurgical diagnosis was transverse myelitis.

### DISCUSSION

Vascular compression of the duodenum has received many names, including superior mesenteric artery syndrome (SMAS), cast syndrome, Wilkie's syndrome and chronic duodenal ileus. A post-mortem description by von Rokitanski (1861)[1] was followed in 1899 by the first clinical case report.[2] Seventy-five cases were detailed by Wilkie in 1927,[3] since when the syndrome has engendered fluctuating enthusiasm. Some doubt its very existence,[4] but several case reports and reviews of the subject have accrued over the years.[5–8]

Acute and chronic forms of the syndrome have a similar anatomical background. The third part of the duodenum is compressed between the root of the superior mesenteric artery and the aorta or lumbar vertebrae. The angle formed by these structures is normally maintained by a fat pad. In vascular compression of the duodenum this angle may be more acute as a result of severe catabolism and loss of fat, for example after operation,[9] illness, burns,[10] trauma[11] or encasement in a plaster cast[5]. Pronounced lordosis and a short ligament of Treitz may also contribute to the pathogenesis.

Vascular duodenal compression is characterised by postprandial epigastric colic, vomiting and weight loss, which further exacerbates the syndrome.[3] It is commonly relieved by adoption of the prone position.

Barium meal examination during an attack is diagnostic and angiography is rarely indicated.

A conservative approach to management should ordinarily be advised, as obstruction is seldom complete and a number of patients may spontaneously recover. The prone or knee-elbow posture is the position of choice, but in immobile patients a Stryker frame bed may be needed.[11] Parenteral feeding can be employed but, as demonstrated by our second case, enteral feeding with its low morbidity is more appropriate. Under fluoroscopic control and with the aid of a soft-tipped guide wire, the third part of the duodenum was negotiated with ease and safety. The position of the tube in the upper jejunum is maintained by peristalsis.

If conservative measures fail, then the operative treatment of choice is a duodenojejunostomy as proposed by Stavely.[12] Despite its use in *Case 1*, gastrojejunostomy is not recommended because of the risks of stomal ulceration, bile gastritis and the possibility of further duodenal dilatation.[13] Some authors favour division of the ligament of Treitz and relocation of the fourth part of the duodenum to the right of the vertebral column.[7]

### REFERENCES

1. ROKITANSKI, K. (1861) Lehrbuch der Pathologischen Anatomie. Vienna, Braumuller.
2. ALBRECHT, P. A. (1899) Ueber arterio-mesenterialen Darmverschluss an der duodeno-jejunal-greinze und seine ursachliche Beziehung zur Magenerweiterung. *Virchows Arch.* (A) **156**, 285–328.
3. WILKIE, D. P. D. (1927) Chronic duodenal ileus. *Am.J. Med.Sci.* **173**, 643–649.
4. CIMMINO, C. V. (1961) Arteriomesenteric occlusion of the duodenum: an entity? *Radiology* **76**, 828–829.
5. DORPH, M. H. (1950) The cast syndrome. Review of the literature and report of a case. *N.Engl.J.Med.* **243**, 440–442.
6. BARNER, H. B. and SHERMAN, C. D. (1963) Vascular compression of the duodenum. *Int.Abstr.Surg.* **117**, 103–118.
7. AKIN, J. T., GRAY, S. W. and SKANDALAKIS, J. E. (1976) Vascular compression of the duodenum: presentation of ten cases and review of the literature. *Surgery* **79**, 515–522.
8. LUNDULL, L. and THULIN, A. (1980) Wilkie's syndrome – a rarity? *Br.J.Surg.* **67**, 604–606.
9. PURANIK, S. R., KEISER, R. P. and GILBERT, M. G. (1972) Arteriomesenteric duodenal compression in children. *Am.J.Surg.* **124**, 334–339.
10. WALLACE, R. G. and HOWARD, W. B. (1970) Acute superior mesenteric artery syndrome in the severely burned patient. *Radiology* **94**, 307–310.
11. WAYNE, E., MILLER, R. E. and EISEMAN, B. (1971) Duodenal obstruction by the superior mesenteric artery in bedridden combat casualties. *Ann.Surg.* **174**, 339–345.
12. STAVELY, A. L. (1910) Chronic gastromesenteric ileus. *Surg.Gynecol.Obstet.* **11**, 288–297.
13. JONES, S. A., CARTER, R., SMITH, L. L. and JOERGENSON, E. J. (1960) Arteriomesenteric duodenal compression. *Am.J.Surg.* **100**, 262–277.

## Figures and Tables

**Figure 1 f1:**